# Study on the Wear Performance of 20CrMnTi Gear Steel with Different Penetration Gradient Positions

**DOI:** 10.3390/ma18153685

**Published:** 2025-08-06

**Authors:** Yingtao Zhang, Shaokui Wei, Wuxin Yang, Jiajian Guan, Gong Li

**Affiliations:** 1 College of Mechanical & Electrical Engineering, Hohai University, Changzhou 213200, China; weishaokui@foxmail.com; 2 Department of Chemical and Materials Engineering, University of Auckland, Auckland 1010, New Zealand; wuxin.yang@auckland.ac.nz (W.Y.); jgua850@aucklanduni.ac.nz (J.G.); gongli@ysu.edu.cn (G.L.); 3 State Key Laboratory of Metastable Materials Science and Technology, Yanshan University, Qinhuangdao 066004, China

**Keywords:** heat treatment, wear properties, carbon concentration, hardness, 20CrMnTi

## Abstract

This study investigates the wear performance of 20CrMnTi steel, a commonly used material for spiral bevel gears, after heat treatment, with a focus on the microstructural evolution and wear behavior in both the surface and gradient direction of the carburized layer. The results show that the microstructure composition in the gradient direction of the carburized layer gradually transitions from martensite and residual austenite to a martensite–bainite mixed structure, and eventually transforms to fully bainitic in the matrix. With the extension of carburizing time, both the effective carburized layer depth and the hardened layer depth significantly increase. Wear track morphology analysis reveals that the wear track depth gradually becomes shallower and narrower, and the wear rate increases significantly with increasing load. However, the friction coefficient shows little sensitivity to changes in carburizing time and load. Further investigations show that as the carburized layer depth increases, the carbon concentration and hardness of the samples gradually decrease, resulting in an increase in the average wear rate and a progressive worsening of wear severity. After the wear tests, different depths of plowing grooves, spalling, and fish-scale-like features were observed in the wear regions. Additionally, with the increase in load and carburized layer depth, both the width and depth of the wear tracks significantly increased. The research results provide a theoretical basis for optimizing the surface carburizing process of 20CrMnTi steel and improving its wear resistance.

## 1. Introduction

20CrMnTi steel is a typical low-alloy steel that exhibits excellent hardenability, processability, and affordability. It is commonly used in the manufacture of gear parts [1,2,3]. However, under working loads, gears must withstand heavy loads and repeated impacts, which can lead to failure. As modern industrial fields develop, drive systems require higher rotational speeds, causing gears to inevitably experience wear. This wear reduces equipment precision and efficiency, and also generates intermittent noise and vibration [4,5,6]. Therefore, in-depth research on gear wear characteristics is essential for optimizing gear design.

Previous studies have investigated the impact of gear wear on the dynamic characteristics of gear transmission systems by developing dynamic prediction models. It has been found that as wear increases, both gear transmission error and vibration displacement rise, with increasingly intense changes [7]. Under low-speed, heavy-load conditions, early gear wear is governed by the contact fatigue strength of the gear surface. Therefore, surface treatments can be applied to gears to enhance their strength and wear resistance. Various surface treatment methods, such as carburizing, nitriding, shot peening, high-energy beam strengthening, and thermal spraying, have been developed to improve part strength [8,9,10,11,12].

Carburizing heat treatment is a crucial component of the gear manufacturing process and plays a decisive role in ensuring gear performance. Currently, surface carburizing is primarily used to enhance part performance.

The process involves introducing carbon into the material’s surface in a high-temperature environment to form a high-carbon layer, while preserving the original composition in the core. Quenching and tempering are then performed to enhance the gear’s overall performance [13,14]. Heat treatment induces changes in the internal microstructure of materials. Variations in heat treatment parameters result in different internal microstructures, which in turn affect gear performance [15]. Moreover, varying microstructures can lead to different degrees of deformation at different positions on the gear. These deformations increase pitch and tooth orientation errors, reduce accuracy, lower load-carrying capacity, increase meshing noise, and shorten service life [16,17,18], thereby compromising the normal operation of mechanical systems.

Therefore, selecting appropriate gear heat treatment parameters is crucial for ensuring both gear performance and accuracy.

Ensuring the high precision of gears’ post-heat treatment requires controlling the deformation caused by the heat treatment process. Currently, this deformation is primarily addressed through finishing operations. Researchers have studied the correlation between cutting parameters and gear tooth profile errors, adjusting machining parameters to control tooth profile deformation post-heat treatment [18]. Common finishing processes for gears include gear grinding, shaving, planning, honing, and roll shaving [19]. Finishing improves surface quality, which helps reduce noise and wear during operation [20]. However, traditional finishing processes have their drawbacks. For example, grinding wheels can generate heat, potentially causing burn marks on gears. Additionally, honing is time-consuming and does not effectively control gear geometric precision [21]. Moreover, uneven deformation from heat treatment causes varying machining allowances at different locations on the gear, leading to differing carbon concentrations on tooth engagement surfaces, which can affect hardness and wear resistance.

To address the impact of carburizing heat treatment on performance, this study investigates its effects on 20CrMnTi gear steel, focusing on the influence of hardness and carbon concentration on the microstructure and wear resistance of the carburized layer at different depths and under varying heat treatment conditions. Samples from different positions of 20CrMnTi gears were taken using wire cutting. Carbon concentration tests, microstructure observations, and wear property tests were then performed.

## 2. Materials and Methods

### 2.1. Heat Treatment Process

**Figure 1:**
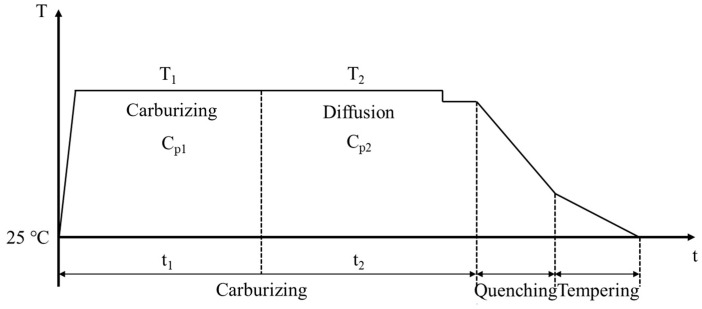
Carburizing and quenching process curve.

The gear material is 20CrMnTi low-carbon alloy steel, with its composition shown in Table 1. Three carburizing and quenching tests with different process parameters were conducted based on the enterprise’s actual production requirements. The sample used was an actual gear-cutting block. The carburizing process curve is shown in Figure 1. The process flow of the three tests is the same, but the process parameters for carburizing and diffusion differ, as shown in Table 2.

**Figure 2:**
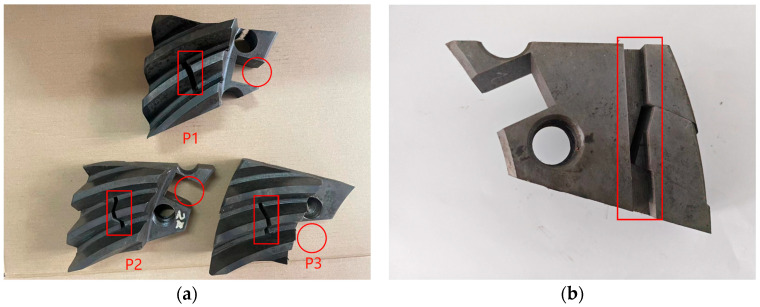
Gear test block sampling location graph: (**a**) microstructure and carbon concentration sample sampling area; (**b**) wear sample sampling area.

To enhance the practical significance and ease of operation of the test results, samples were taken from different locations of the gear test piece after heat treatment. The specific sampling locations are shown in Figure 2: (1) the gear tooth cross-section within the red box area in Figure 2a for microstructure analysis; (2) the gear test block step within the circular area in Figure 2a for carbon concentration analysis; and (3) the bottom of the gear test block within the red box area in Figure 2b for wear testing.

### 2.2. Carbon Concentration Test

**Figure 3:**
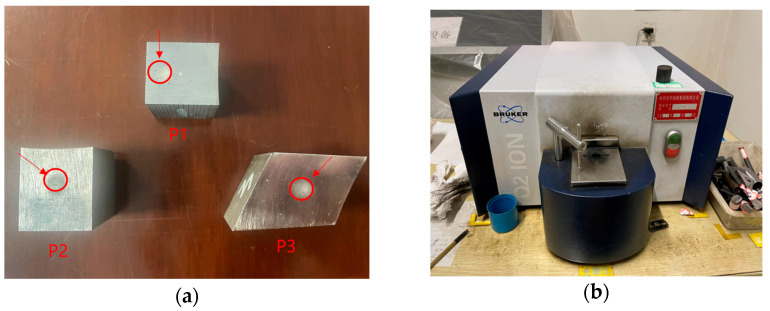
(**a**) 20CrMnTi carbon concentration test sample; (**b**) direct-reading spectrometer.

The furnace gear test block was sampled at the step position, and the resulting sample is shown in Figure 3a. The oxidized layer was removed from the specimen, and the carbon concentration was measured using a German BRUKER Q2 ION direct-reading spectrometer (Karlsruhe, Germany) (Figure 3b). The carbon concentration gradient test was conducted for the three different processes in Table 2. First, the carbon concentration of the surface layer was measured, followed by grinding off the measurement marks using a grinder. The grinding depth was then measured with a micrometer. These steps were repeated until the substrate was reached.

### 2.3. Microstructure and Hardness Test

**Figure 4:**
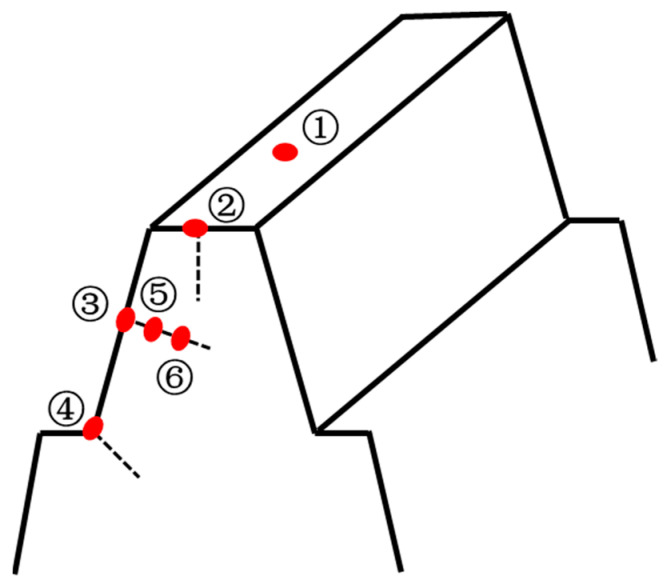
Microstructure and microhardness test position diagram.

The gear test blocks, prepared using wire-cutting technology, were subjected to different heat treatment process conditions. The sample surface was polished sequentially with sandpaper of increasing mesh size, then mechanically polished to a mirror finish, and finally cleaned with alcohol. The surface was then corroded using a nitric acid solution for 6 s. Immediately after corrosion, the surface was cleaned with ethanol and dried with a hair dryer. The microstructure of the test pieces was observed using a 6XB-PC metallurgical microscope (Shanghai Yongheng, Shanghai, China). The metallographic test positions are shown in Figure 4: position 1 corresponds to the tooth top surface; positions 2–4 correspond to the tooth top, indexing circle, and tooth root surfaces, respectively; positions 5 and 6 correspond to the middle of the carburized layer at the indexing circle and the gear substrate. The microhardness was tested using an HVS-1000A hardness tester (Laizhou Wenchang Runyuan Testing Instrument Factory, Laizhou, China). The test position was perpendicular to the surfaces of 2, 3, and 4, extending to the depth of the carburized layer. The test load was 500 g, and the loading time was 15 s.

### 2.4. Wear Test

**Figure 5:**
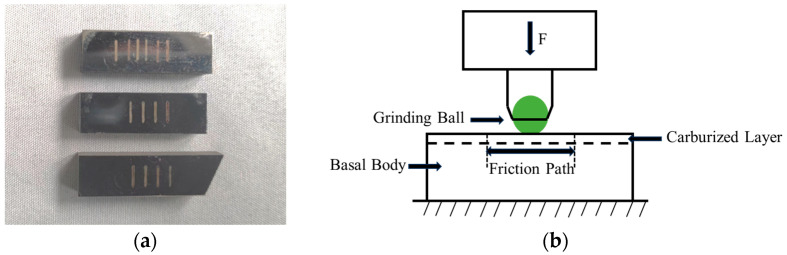
(**a**) Wear test samples; (**b**) schematic diagram of the wear experiment.

In contrast to the carbon concentration and metallographic specimens, the wear specimens were sampled from the bottom surface of the gears, as shown in Figure 5a. The wear principle is illustrated in Figure 5b. Reciprocating sliding friction wear experiments were conducted using the CFT-1 type comprehensive tester (Zhongke Kaihua, Lanzhou, China) for surface properties at room temperature under the following conditions: loads of 30 N, 50 N, 70 N, and 90 N, an experimental time of 120 min, a reciprocating length of 5 mm, a sampling frequency of 1 Hz, a total stroke of 120 m, and a rotational speed of 100 rpm. YG6 tungsten steel balls, with a diameter of 6 mm and a hardness of 92 HRA, were used as the friction counterpart.

To study the effect of carbon concentration on the wear resistance of the specimen perpendicular to the carburized layer, wear experiments were first conducted on the surface of the outermost carburized layer. Then, a grinding machine was used to remove a specific thickness, which was measured with a micrometer. After grinding with sandpaper and polishing, wear tests were performed on the next layer. This process was repeated, and experiments were stopped when the microhardness of the carburized layer fell below the hardness of the effective hardened layer (550 HV). The thickness of the material worn off from each layer, corresponding to the three processes, was 0.3 mm, 0.2 mm, and 0.4 mm, respectively, depending on the depth of the carburized layer. After each wear experiment, the wear volume of the specimens was determined using the surface profiler integrated with the wear testing machine. A total of five sets of experimental data were measured, with each measurement interval being approximately 1 mm. The average wear volume, *V* (mm^3^), was calculated as the mean value of these measurements. The wear rate was then calculated using Equation (1).

(1) $$WL=\frac{V}{F\times L}$$

where *WL* is the average wear rate of the specimen (mm^3^·N^−1^·m^−1^), *V* is the average wear volume of the specimen (mm^3^), *L* is the total experimental reciprocating stroke (m), and *F* is the experimental applied normal load (N).

## 3. Results and Discussions

### 3.1. Carbon Concentration Gradient

**Figure 6:**
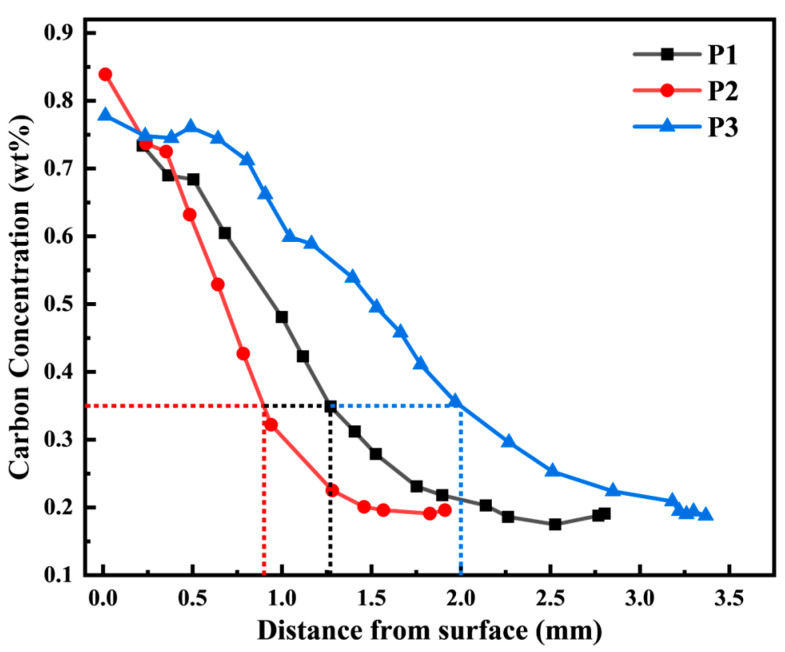
Carbon concentration gradient curve of 20CrMnTi carburized layer.

As shown in Figure 6 the carbon concentration gradient of the gear specimens after three different carburizing processes is displayed. In the inner area of each specimen, 2 mm away from the surface, a carburized layer is formed with a decreasing carbon concentration from high to low. The effective carburized layer depths obtained from the three processes are different: 1.27 mm, 0.92 mm, and 1.96 mm, respectively. This variation is due to the carburizing time; the longer the carburizing time, the deeper the effective carburized layer. The carbon concentration gradient curves of all three processes show a slight “low head” phenomenon, which may be caused by unstable carbon potential in the furnace during the carburizing process or slight decarburization on the surface of the specimen.

### 3.2. Microstructure and Hardness

**Figure 7:**
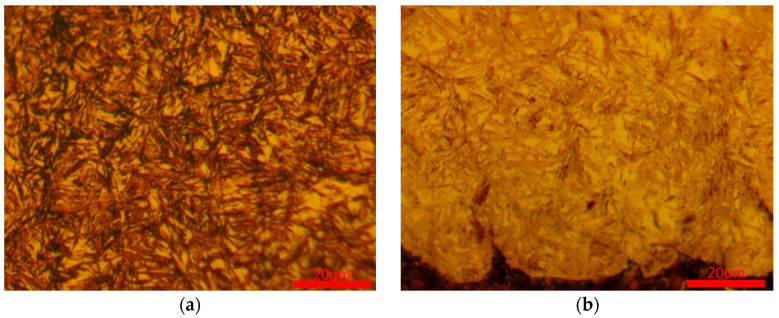

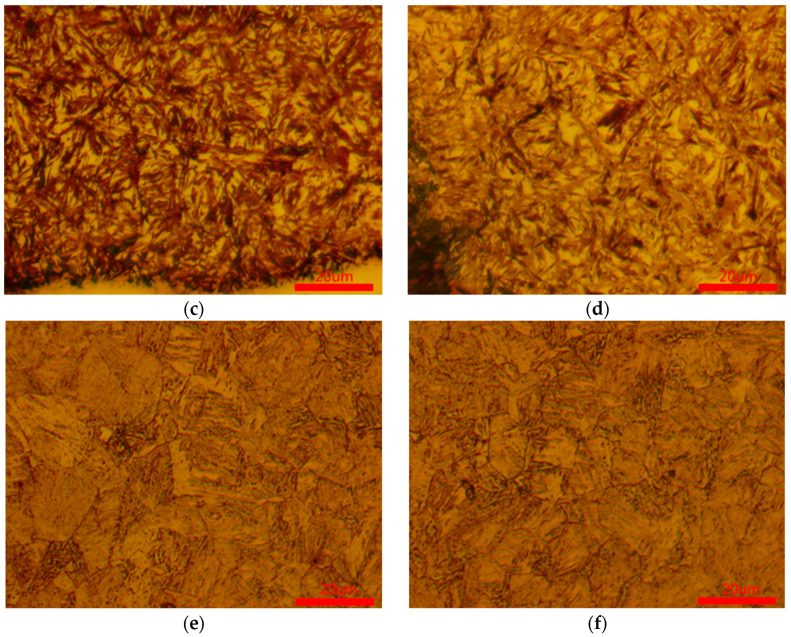
Metallographic micrographs of spiral bevel gears at six different positions: (**a**) position 1; (**b**) position 2; (**c**) position 3; (**d**) position 4; (**e**) position 5; (**f**) position 6.

Figure 7 corresponding to Figure 4, metallographic micrographs of the spiral bevel gear at six different positions. Figure 7a shows the metallographic structure of the tooth top surface, while Figure 7b–d show the metallographic structures of the tooth top, pitch circle, and root section surfaces, respectively. The surface of the carburized layer is mainly composed of martensite and contains a small amount of residual austenite. Moving downward from the surface of the carburized layer, the microstructure gradually transitions from martensite to a mixed structure of martensite and lamellar bainite, and finally to bainite, as shown in Figure 7e,f.

**Figure 8:**
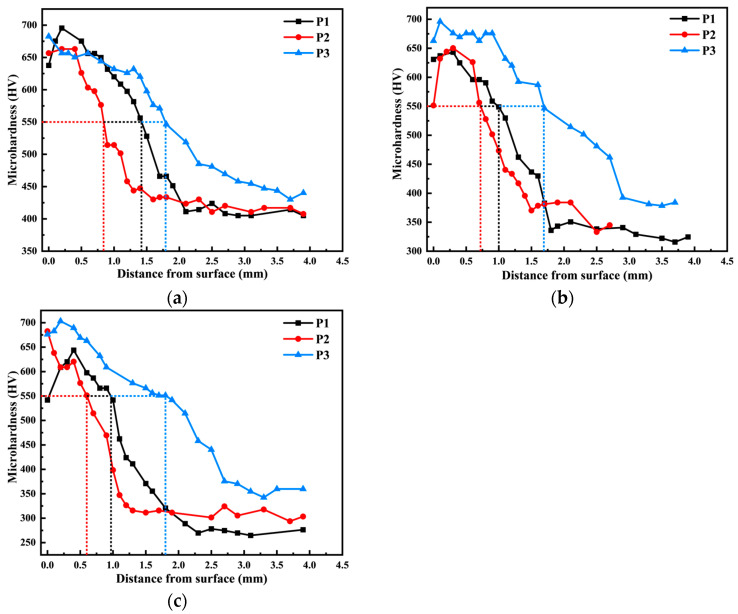
Hardness gradient profiles of three process samples at different positions: (**a**) tooth top; (**b**) indexing circle; (**c**) tooth root.

Figure 8 illustrates the hardness gradient curves of 20CrMnTi carburized samples at various locations under P1, P2, and P3 conditions. As the carbon content decreases from the surface to the center, the hardness of the samples decreases accordingly for all three processes. Internal oxidation occurs on the carburized layer surface due to decarburization, which results in a decrease in both carbon concentration and microhardness at the surface of the carburized layer. Under the P1, P2, and P3 conditions, the effective depth of the hardened layer at the tooth top is 1.42 mm, 0.84 mm, and 1.79 mm, respectively; at the index circle, it is 1.00 mm, 0.72 mm, and 1.70 mm, respectively; and at the tooth root, it is 0.97 mm, 0.60 mm, and 1.80 mm, respectively. The carburizing time follows the order of P3 > P1 > P2, and consequently, the depth of the effective hardened layer at each position of the gear follows the same order. This indicates that the depth of the effective hardened layer increases with a longer carburizing time.

### 3.3. Carburized Layer Surface Wear Performance Analysis

#### 3.3.1. Coefficient of Friction

**Figure 9:**
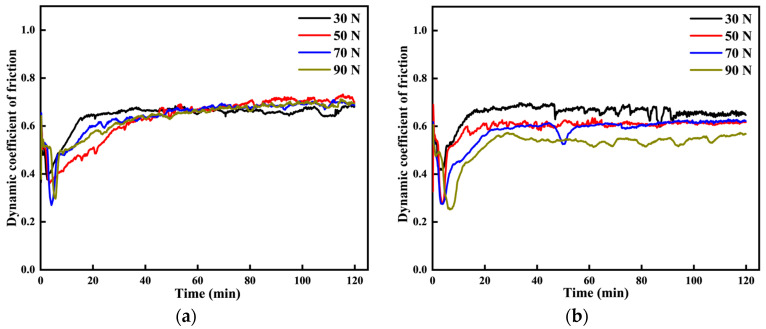

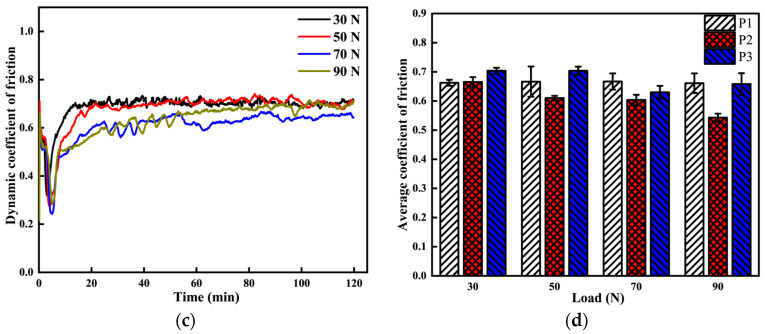
Surface friction coefficients of carburized layers of 20CrMnTi under different processes and loads: (**a**) surface coefficient of friction of P1 carburized layer; (**b**) surface coefficient of friction of P2 carburized layer; (**c**) surface coefficient of friction of P3 carburized layer; (**d**) average coefficient of friction of P1, P2, and P3 carburized layer surfaces.

Figure 9 presents the dynamic and average friction coefficients of the 20CrMnTi carburized layer surface under different processes and loads. As shown in the figure, the friction coefficients initially rise rapidly, then decline, and eventually stabilize. This process includes the friction and stabilization phases. In the initial stage of friction, the rough surface of the specimen causes uneven contact, concentrating the load at specific points and forming local spot welding. As the reciprocating motion continues, the spot weld ruptures, leading to a rapid increase in the friction coefficient for each group. The combined effect of contact and shear stresses during friction causes plastic deformation on the specimen surface, resulting in work hardening. Work hardening increases the material’s hardness and brittleness, causing it to gradually break off as wear chips during continued friction. The generated wear debris enters between the friction surfaces, temporarily separating them and reducing the friction coefficient. As the wear experiment progresses, the generation and discharge of wear debris reach a dynamic equilibrium, causing the friction coefficient to gradually stabilize.

To minimize errors, the average friction coefficients are calculated by averaging the dynamic friction coefficients during the stabilization period, from the 20th minute to the end of the test. As shown in Figure 9d, the average coefficient of friction for the P1 specimen fluctuates between 0.66 and 0.67 across the four loads, with a minimal effect of load on the friction coefficient. The average coefficient of friction for the P2 specimen fluctuates between 0.54 and 0.67, decreasing as the load increases. According to modern friction theory, an increase in normal load enlarges the actual contact area, thereby reducing the friction coefficient [22]. The average coefficient of friction for the P2 specimen fluctuates between 0.54 and 0.67, decreasing as the load increases. According to modern friction theory, an increase in normal load enlarges the actual contact area, thereby reducing the friction coefficient. As the load increases, the micro-convex bodies are compressed and deformed, increasing the contact area and reducing the friction coefficient. When the load reaches a critical point, the micro-convex bodies undergo shear fracture, accompanied by the plastic deformation of the material and adhesive detachment, resulting in an increase in the friction coefficient.

#### 3.3.2. Wear Volume Loss

**Figure 10:**
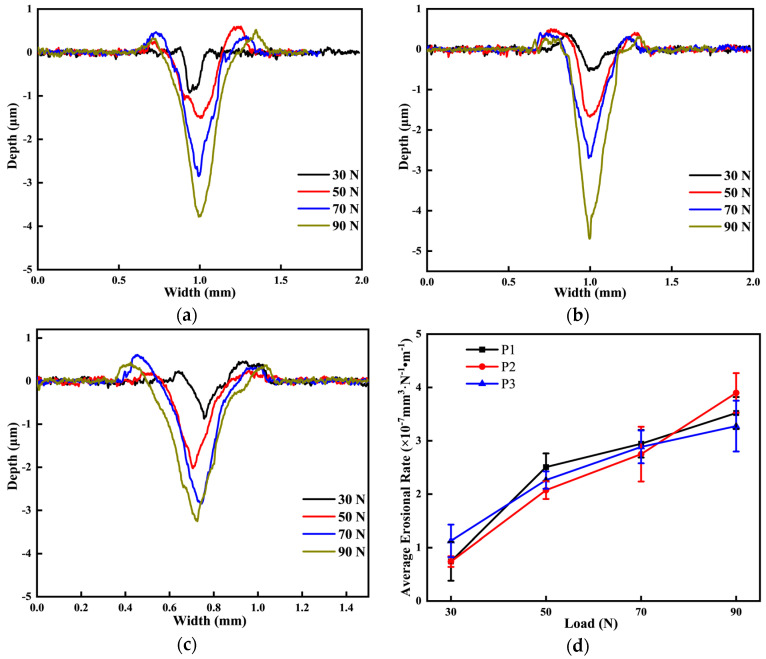
Surface wear characteristics of the carburized layer of 20CrMnTi under different processes and loads: (**a**) surface abrasion morphology of the carburized layer of P1; (**b**) surface abrasion morphology of the carburized layer of P2; (**c**) surface abrasion morphology of the carburized layer of P3; (**d**) average wear rate of the carburized layer of P1, P2, P3.

Figure 10 shows the cross-sectional profile and average wear rate of the wear marks on the surface of the 20CrMnTi carburized layer. Figure 10a–c present the two-dimensional morphology of the abrasion marks for the P1, P2, and P3 specimens under varying loads. It can be observed that both the width and depth of the abrasion marks increase with the applied load. Under a 90 N load, the P2 specimen shows the greatest abrasion depth of 4.7 μm, followed by the P1 specimen with 3.78 μm. The P3 specimen has the shallowest abrasion depth, measuring 3.25 μm. The greater depth and width of the abrasion marks indicate poorer abrasion resistance. The hardness results in Figure 8 demonstrate that hardness is a key factor in the wear resistance of the specimens under the three carburizing processes. Higher hardness correlates with better wear resistance. As the load increases, the average wear rate of the specimens also rises. This is because the increased vertical load causes the friction ball to penetrate deeper into the specimen, resulting in greater wear due to the tangential force.

#### 3.3.3. Wear Mechanism

**Figure 11:**
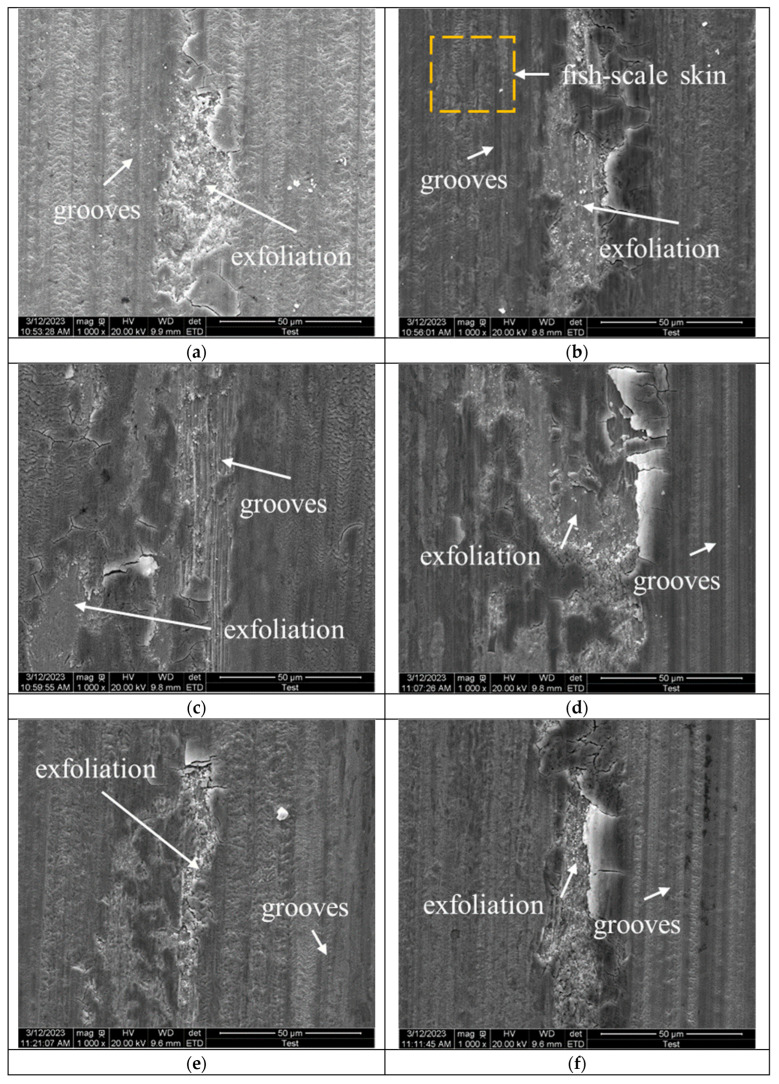
SEM micrographs of surface wear of carburized layer of 20CrMnTi under different processes and loads: (**a**) P1-30 N; (**b**) P1-90 N; (**c**) P2-30 N; (**d**) P2-90 N; (**e**) P3-30 N; (**f**) P3-90 N.

As shown in Figure 11, there are significant differences in the wear track morphology and average wear rate of the samples under 30 N and 90 N load conditions. To facilitate the analysis and distinguish the effect of loading on the wear mechanism, the wear morphology on the carburized layer surface of the samples under 30 N and 90 N load conditions was selected for investigation. The figure shows furrows, a typical characteristic of abrasive wear, as shown in Figure 11. And as the load increases, the depth of the furrows becomes progressively greater. Fretting wear occurs during the grinding process between the friction materials. When the friction material embeds into the substrate, carbides within the substrate detach and act as hard particles that damage the surface, forming noticeable furrows. Figure 11b clearly shows a fish-scale pattern, a characteristic of fatigue wear. It also shows noticeable flaking, caused by heat during grinding, which leads to the formation of oxides on the substrate. These oxides then continue to flake during friction. This results in the adhesive and oxidative wear of the material. However, since the wear test is performed on the surface of the carburized layer, the surface microstructure is primarily composed of martensite and residual austenite. The microhardness of the contact material is also high. This means that particularly exaggerated wear characteristics, such as deeper grooves and obvious exfoliation, do not occur.

### 3.4. Analysis of Wear Performance at Different Gradient Positions of the Carburized Layer

**Figure 12:**
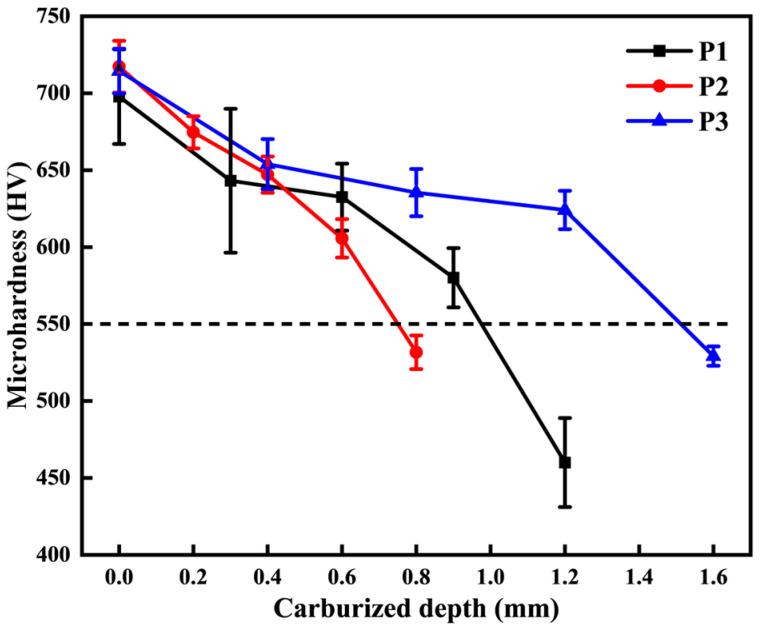
Microhardness gradient of different carburized layer positions.

The wear samples were stripped layer by layer using a grinding method. Due to differences in carburized layer depth under different processes, the thickness of each stripped layer for the P1, P2, and P3 samples was 0.3 mm, 0.2 mm, and 0.4 mm, respectively. After grinding, the thickness of each layer, relative to the initial surface, was recorded. Simultaneously, the microhardness of each layer was measured, and the results are shown in Figure 12. As the number of layers increases, the wear surface moves further from the carburized layer, leading to a gradual decrease in both carbon concentration and microhardness. In the fifth layer, the microhardness of the worn surface is lower than the hardness of the effective hardened layer (550 HV), significantly decreasing compared to the first four layers. It is expected that wear of the fifth layer will be more severe than that of the first four layers.

#### 3.4.1. Coefficient of Friction

**Figure 13:**
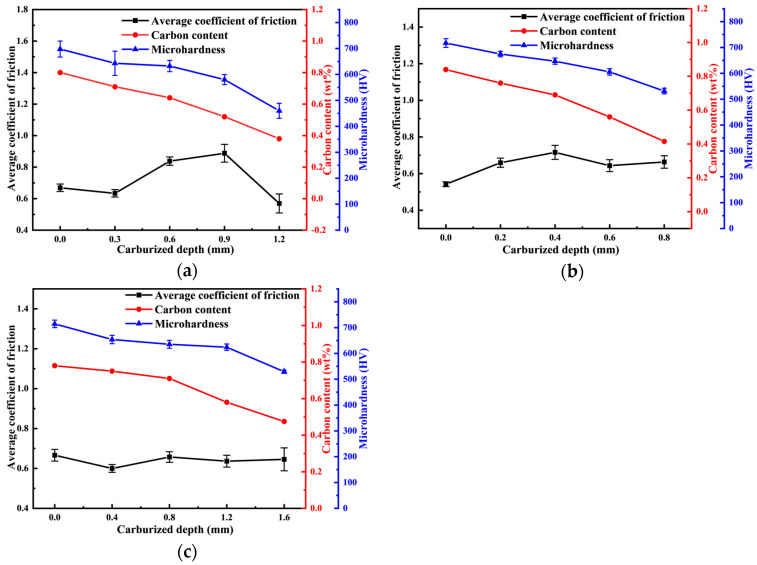
The influence of the depth of the carburized layer on the average friction coefficient, carbon concentration, and microhardness of the worn surface of samples under different process conditions: (**a**) P1; (**b**) P2; (**c**) P3.

As the depth of the carburized layer increases, the carbon concentration and microhardness of the wear surface of the samples gradually decrease. However, the average coefficient of friction of the samples does not exhibit a consistent increasing or decreasing trend under the three process conditions. This phenomenon suggests that the carbon concentration and hardness of the wear surface of the specimens have a limited effect on the average coefficient of friction. Figure 13 demonstrates the trends of carbon concentration, hardness distribution, and the average friction coefficient of the samples as the depth of the carburized layer increases for the three different process conditions.

#### 3.4.2. Wear Volume

**Figure 14:**
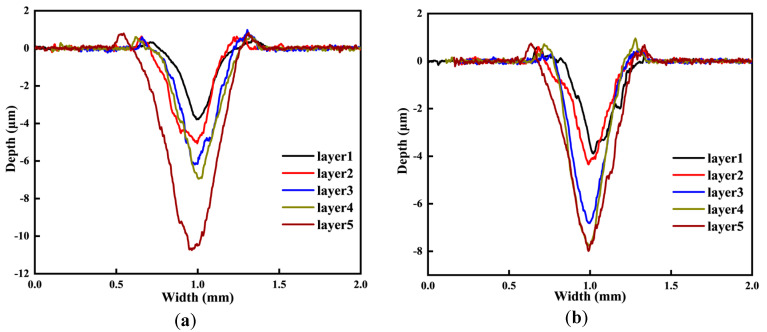

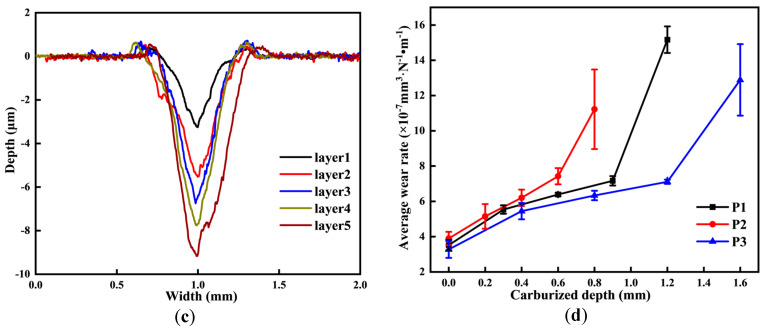
Morphology of wear marks at different carburized layer locations: (**a**) P1; (**b**) P2; (**c**) P3; (**d**) average wear rate.

As the number of layers peeled off from the carburized layer of the gear increases, the depth of the wear surface from the carburized layer also increases. Consequently, both the depth and width of the wear marks observed in the wear experiments gradually increase. As shown in Figure 14d below, the average wear rate of the specimen also increases. This phenomenon occurs because, as the depth of the carburized layer increases, the carbon concentration of the wear surface decreases, along with a reduction in hardness. These changes lead to the more severe adhesive wear of the specimen. The shedding of debris results in an increased number of deep craters, while abrasive wear further contributes to the formation of furrow grooves. These factors collectively lead to greater wear loss, ultimately increasing both the depth and width of the wear marks.

**Figure 15:**
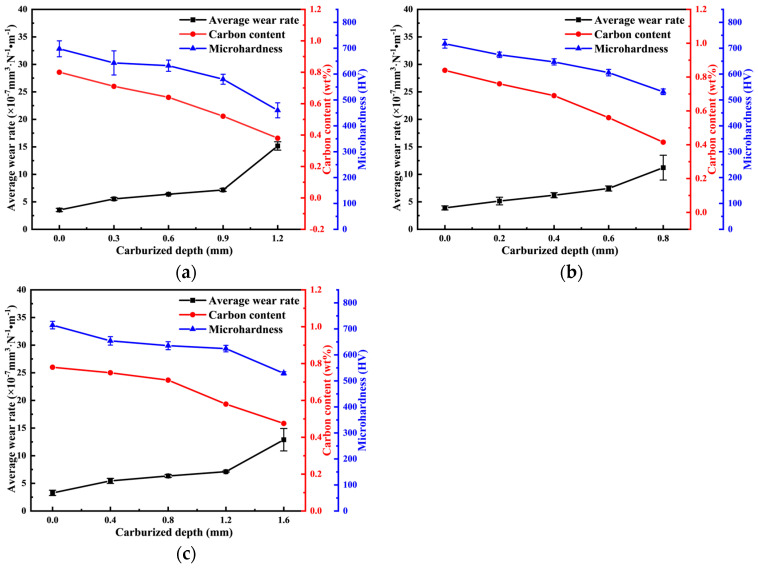
The influence of the depth of the carburized layer on the average wear rate, carbon concentration, and microhardness of the worn surface of samples under different process conditions: (**a**) P1; (**b**) P2; (**c**) P3.

As the depth of the carburized layer increases, the carbon concentration and microhardness of the sample’s wear surface decrease under all three process conditions. However, the average wear rate increases consistently, showing an inverse relationship with the carbon concentration and hardness. This suggests that the carbon concentration and microhardness of the sample surface significantly influence its wear performance. Specifically, a lower carbon concentration and hardness lead to higher wear rates and worse wear resistance [23]. Figure 15 illustrates the trends in carbon concentration, hardness distribution, and average wear rate as the carburized layer depth increases under the three different process conditions.

**Figure 16:**
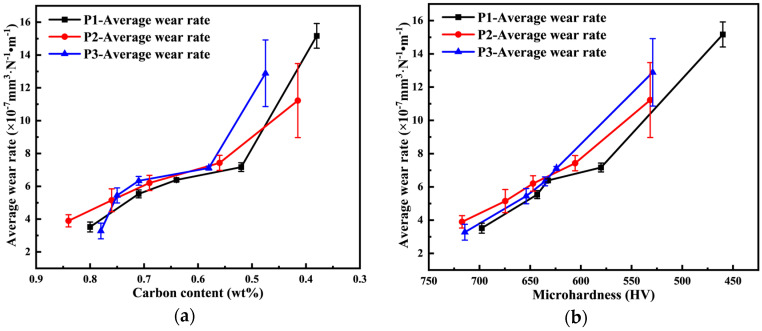
Effect of different process conditions on the average wear rate of specimen surface: (**a**) carbon concentration; (**b**) microhardness.

Based on the above observations, the tissue distribution results indicate that the carburized layer surface consists primarily of high-carbon martensite with high hardness. As the carburized layer depth increases, the carbon concentration decreases, and the tissue transitions to primarily low-carbon martensite and lamellar bainite with a lower hardness. Near the substrate, the tissue predominantly becomes bainite with a low hardness, which is consistent with the results shown in Figure 14. The carbon concentration on the wear surface of the specimen decreases as the carburized layer depth increases. Both the tissue and carbon concentration of the specimen simultaneously influence the microhardness of the wear surface, leading to a decrease in hardness and a corresponding reduction in the specimen’s resistance to wear. As a result, the average wear rate of the specimen increases as the carbon concentration and microhardness decrease. Figure 16 illustrates the trend in the average wear rate as the surface carbon concentration and microhardness decrease under different process conditions.

#### 3.4.3. Wear Mechanism

**Figure 17:**
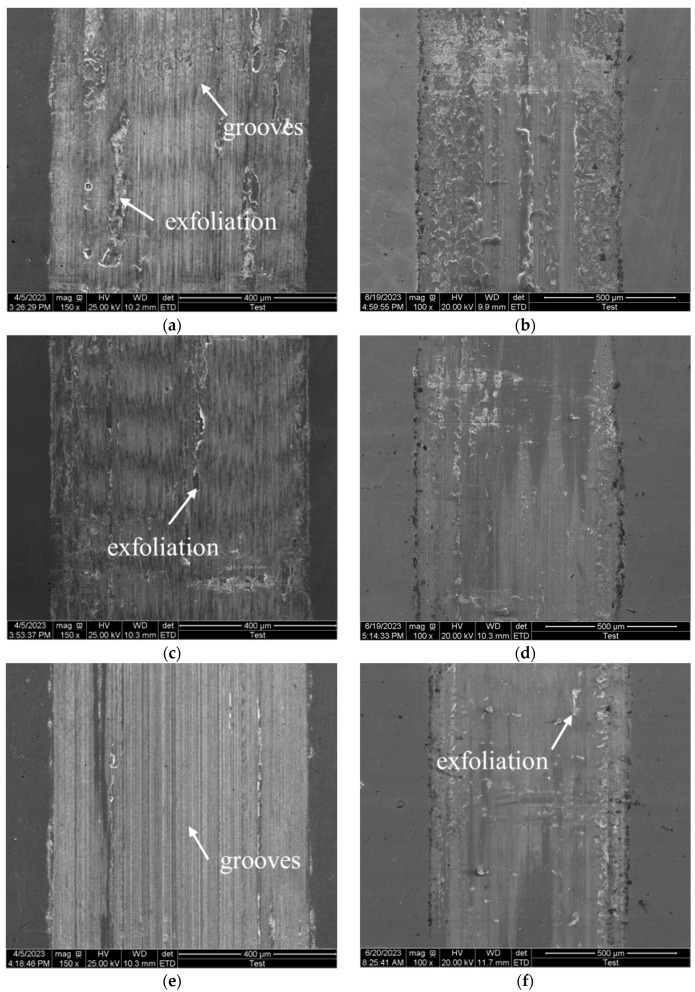
Wear patterns of the specimens under a 90 N load: (**a**) P1 second layer; (**b**) P1 fifth layer; (**c**) P2 second layer; (**d**) P2 fifth layer; (**e**) P3 second layer; (**f**) P3 fifth layer.

The wear morphology of the carburized layers of the second and fifth layers under 90 N loading conditions is analyzed, as shown in Figure 17. Figure 17a–e show the wear morphology of the second layer at 150 × magnification, while Figure 17b,d,f show the wear morphology of the fifth layer at 100 × magnification. From a macroscopic perspective, compared to the second layer, the fifth layer exhibits deeper wear marks and a dark-colored abrasive chip accumulation zone. This is due to the lower carbon concentration of the fifth layer’s wear surface, which results in reduced hardness and more severe abrasive wear [24]. The abrasive chips formed during wear accumulate on the wear surface, eventually creating accumulation bands on both sides of the wear scar. The figure below shows that there is no noticeable accumulation zone on either side of the wear mark in the second layer, indicating that the carbon concentration and hardness of the wear surface are decisive factors in the sample’s wear behavior.

Additionally, exfoliation was observed in the wear morphology of both the second and fifth layers, primarily due to adhesive wear. Compared to the second layer, the fifth layer has a lower surface hardness, so the friction ball, with a higher hardness, transfers part of the material during adhesive wear. As the friction ball continues to slide, the transferred material gradually detaches from the friction ball due to fracture or fatigue and remains in the wear marks, forming new abrasive particles. This results in more severe abrasive wear.

## 4. Conclusions

In this paper, the effects of load, carbon concentration and hardness on the wear behavior of 20CrMnTi gear steel are investigated from the aspects of microstructure, friction coefficient, average wear rate, and wear mechanism. The results conclude that the microstructure of 20CrMnTi steel undergoes a transformation along the depth of the carburization layer direction, initially consisting of martensite and residual austenite, then evolving into a mixed martensite-bainite structure, and finally becoming entirely bainitic at the matrix. Based on the results of the wear test, it can be seen that the martensite hardness is higher than the bainite, so the wear resistance of the material deteriorates as the martensite content decreases.

This microstructural evolution significantly affects the material properties. Both the effective depth of the penetration layer and the hardened layer depth increased significantly with longer carburizing times. This suggests that carburizing time plays a crucial role in the formation of the carburized layer and enhances surface hardness. Morphological analysis of the abrasion marks revealed that their depth and width decreased with increasing carburizing time. Simultaneously, the wear rate increased significantly with increasing load. Although the wear rate is strongly influenced by load changes; the friction coefficient remains insensitive to both carburizing time and load variations, as the carburized layer depth increases, carbon concentration and hardness decrease, resulting in a higher average wear rate and increased wear degree. This suggests a negative correlation between carburized layer depth and material wear resistance. After the wear test, features such as furrows, spalling, and fish-scale-like skin of varying depths appeared in the wear area, highlighting the effects of load and carburized layer depth on the wear mechanism. The width and depth of wear marks increased significantly with a higher load and greater carburized layer depth, suggesting that deeper carburized layers may lead to more severe wear.

The results indicate that carburizing time effectively increases the carburized layer’s depth. Further analysis reveals that the wear resistance of 20CrMnTi gear steel in the carburized layer depth direction is primarily governed by the surface carbon concentration and hardness. This study provides a foundation and new insights for optimizing subsequent gear manufacturing and processing.

## Figures and Tables

**Table 1:** 20CrMnTi gear steel composition.

Elements	C	Si	Mn	Cr	Ni	Ti	P	S	Cu	Fe
Wt%	0.21	0.24	0.9	1.18	0.15	0.04	0.015	0.0079	0.081	Bal

**Table 2:** Different carburizing process solutions for 20CrMnTi gear steel.

Carburization Process	Carburizing			Diffusion		
	Temp T_1_ (°C)	Time t_1_ (min)	Carbon Potential C_p1_ (%)	Temp T_2_ (°C)	Time t_2_ (min)	Carbon Potential C_p2_ (%)
P1	910	420	1.2	910	150	0.8
P2	910	210	1.2	910	60	0.9
P3	920	870	1.2	920	360	0.8

## Data Availability

The original contributions presented in the study are included in the article. Further inquiries can be directed to the corresponding author.
